# Gastrointestinal Dysfunction in Parkinson’s Disease

**DOI:** 10.3390/jcm10030493

**Published:** 2021-01-31

**Authors:** Casper Skjærbæk, Karoline Knudsen, Jacob Horsager, Per Borghammer

**Affiliations:** Department of Nuclear Medicine & PET, Aarhus University Hospital, Palle Juul-Jensens Boulevard 165, J220, 8200 Aarhus N, Denmark; karoknud@rm.dk (K.K.); jacobnls@rm.dk (J.H.); perborgh@rm.dk (P.B.)

**Keywords:** Parkinson’s disease, autonomic, gastrointestinal, constipation, alpha-synuclein, parasympathetic

## Abstract

Parkinson’s disease (PD) is the second most common neurodegenerative disease. Patients show deposits of pathological, aggregated α-synuclein not only in the brain but throughout almost the entire length of the digestive tract. This gives rise to non-motor symptoms particularly within the gastrointestinal tract and patients experience a wide range of frequent and burdensome symptoms such as dysphagia, bloating, and constipation. Recent evidence suggests that progressive accumulation of gastrointestinal pathology is underway several years before a clinical diagnosis of PD. Notably, constipation has been shown to increase the risk of developing PD and in contrast, truncal vagotomy seems to decrease the risk of PD. Animal models have demonstrated gut-to-brain spreading of pathological α-synuclein and it is currently being intensely studied whether PD begins in the gut of some patients. Gastrointestinal symptoms in PD have been investigated by the use of several different questionnaires. However, there is limited correspondence between subjective gastrointestinal symptoms and objective dysfunction along the gastrointestinal tract, and often the magnitude of dysfunction is underestimated by the use of questionnaires. Therefore, objective measures are important tools to clarify the degree of dysfunction in future studies of PD. Here, we summarize the types and prevalence of subjective gastrointestinal symptoms and objective dysfunction in PD. The potential importance of the gastrointestinal tract in the etiopathogenesis of PD is briefly discussed.

## 1. Introduction

Parkinson’s disease (PD) is the second most common neurodegenerative disease affecting 2–3% of the population above 65 years of age [1]. Slowness of movements (bradykinesia) in combination with rigidity or tremor constitute the motor symptoms necessary for a clinical diagnosis [2] but non-motor symptoms (NMS) are numerous and often burdensome [3].

NMS attributable to the digestive system are particularly common and dysfunction along the entire length of the digestive tract give rise to symptoms such as dysphagia, bloating, early satiety, and constipation [4]. Interestingly, constipation may precede PD by more than a decade [5], supporting the relatively recent hypothesis that PD may in fact originate in the enteric nervous system and spread to the CNS via the vagus nerve [6]. In support, pathological aggregates of α-synuclein have been detected in gastrointestinal tissues removed several years prior to clinical diagnosis of PD [7], and epidemiological studies have shown that truncal vagotomy decreases the risk of PD by 40–50% [8,9]. In addition, injections of preformed α-synuclein fibrils into the gut wall of rodents leads to initiation and gut-to-brain spreading of α-synuclein aggregates in a pattern highly similar to that seen in human patients—and similar findings were seen after exposing the stomach to the pesticide rotenone [10,11].

Therefore, it is of considerable importance to unravel the etiopathogenic role of the gastrointestinal tract in PD, and to improve our understanding and assessment methods of subjective gastrointestinal symptoms and objective gastrointestinal dysfunction.

This review provides a brief summary of gastrointestinal pathophysiology of PD and highlights specific gastrointestinal symptoms and objective measures of dysfunction relevant for further research. The current approaches to treatment of gastrointestinal symptoms in PD will also be briefly touched upon.

## 2. Gastrointestinal Pathology in PD

The loss of dopaminergic neurons in the substantia nigra of the brainstem plays a pivotal role in onset and progression of motor symptoms [2]. The distinctive aggregates of α-synuclein, now termed Lewy pathology (LP), were first identified by Friedrich Lewy in 1912 and since then, the distribution of LP in PD has been extensively studied [12].

In most patients, the dorsal motor nucleus of vagus (DMV) in the medulla oblongata is severely affected by LP with a 50% loss of neurons [13,14,15] and the vagal nerve containing the visceromotor fibers from the DMV also shows involvement [13]. The density of LP in the gastrointestinal tract follows a rostro-caudal gradient corresponding to the density of vagal motor terminals [16] with the lower esophagus and the stomach representing the most affected areas, while the upper esophagus is spared corresponding to its innervation by somatomotor fibers from the relatively unaffected ambiguus nucleus [13,17,18]. Only sparse pathology is found throughout the colon including the distal third of the colon and rectum that are not innervated by the vagal nerve but by fibers from sacral nuclei in which LP is also found [13,17,18]. Notably, this rostro-caudal gradient of pathology is in sharp contrast to the relative magnitude of reported symptoms as constipation and defecatory problems are more prevalent than dysphagia especially in early disease [19,20]. Constipation may present more than a decade prior to clinical diagnosis [5,21].

The link between the gastrointestinal tract and development of PD is also supported by the finding that truncal vagotomy lowers the risk of PD when compared to a super-selective vagotomy in which only a few fibers to the stomach are cut [8,9]. Naturally, these are observational studies, but the idea of retrograde spreading of pathology in PD, as initially postulated Braak et al. [6,22,23], has found additional support in animal models capable of reproducing a formation and spreading of α-synuclein aggregates after injection of either preformed α-synuclein fibrils into the gut wall or by exposing the gut to the pesticide rotenone [10,11].

In vivo studies of human intestinal biopsies have found α-synuclein to be frequent in PD patients compared to controls [24,25,26]. Interestingly, the appendix vermiformis is a hot spot of α-synuclein aggregation in healthy adults [27], but conflicting epidemiological studies of appendectomized individuals’ risk of PD later in life has raised doubts about the importance of appendicular α-synuclein aggregation in the development of PD [28,29,30]. Importantly, the pathological studies have been cross-sectional and do not clarify whether the pathology is spreading from one gastrointestinal hot spot prior to disease as in the aforementioned animal models. Additionally, the specificity and sensitivity of α-synuclein staining may be suboptimal and further limited by insufficient availability of full-thickness gastrointestinal tissue making such human longitudinal in vivo studies difficult [31,32].

The intestinal mucosa, only millimeters away from the enteric nervous system (ENS), is exposed to not only environmental toxins but also potentially toxic microbial metabolites creating high demands for the epithelial barrier, which could be potential trigger factors for initiating PD [33]. Interestingly, exposure to *Escherichia coli* producing the protein curli enhances the aggregation of α-syn in aged Fischer rats [34]. Furthermore, in a transgenic mouse model of PD with overexpression of α-synuclein it was demonstrated that colonization of germ-free mice with microbiota transplants from PD patients enhance the development of physical impairments compared to microbiota transplants from healthy volunteers [35]. Studies of the human microbiome in PD have recently been reviewed elsewhere [36] and although some studies point to interesting differences suggestive of a pro-inflammatory microbiome in PD patients the findings are heterogenous and mainly from cross-sectional studies of manifest PD. Elevated levels of pro-inflammatory markers such as IL-1α have also been found in stool samples when comparing PD patients with controls [37]. Furthermore, levels of zonulin in stool samples were also found to be elevated in PD indication a degradation of intestinal tight junctions in PD [38]. Signs compatible with increased intestinal permeability *(leaky gut)* in PD was demonstrated in a small sample of 9 PD patients and 7 controls. That study found that the gastrointestinal permeability for sucralose, but not lactulose or mannitol, was increased in the PD group [39]. In support of the role for gastrointestinal inflammation in PD is the finding that inflammatory bowel disease increases the risk of PD later in life [40]

Ideally, these hypotheses about the etiology of PD should be tested in longitudinal human studies, but as it is inherently difficult to study the silent onset of pathology this has so far not been possible. However, a peculiar sleep disorder characterized by disruption of the normal atonia during REM-sleep together with dream enactment has gained interest. Nearly all people with this sleep disorder, called REM-sleep behavior disorder (RBD), progresses to manifest PD or the highly similar condition dementia with Lewy bodies (DLB) within 15 years [41]. The disorder arises as a consequence of damage to certain nuclei in the pons and is the strongest prodromal marker of PD [42]. Remarkably, patients with RBD display a greater density of gastrointestinal LP than patients without [43]. Likewise, loss of cardiac sympathetic innervation and colonic acetylcholinesterase in RBD cases have been shown to be comparable to that of diagnosed PD patients, although the dopaminergic system in the RBD cases was still intact [44]. Consequently, it has been proposed that RBD represents a prodromal biomarker of a gastrointestinal, body-first onset of PD [45].

Overall, widespread pathology of the gastrointestinal tract is indeed present already in prodromal stages of PD at least in a considerable fraction of cases. Yet, there are no longitudinal studies in humans to confirm the idea of a gastrointestinal onset of disease, but the relevance of gastrointestinal symptoms and objectives measures of dysfunction is clearly present.

## 3. Gastrointestinal Symptoms in PD

A wide range of NMS in PD arise from the gastrointestinal tract and several questionnaires have been developed to quantify the symptoms including the Scales for Outcomes in Parkinson’s Disease—Autonomic [19,46] (SCOPA-AUT), the Non-Motor Symptoms Scale [47,48] (NMSS) and the Non-motor Symptoms Questionnaire [49] (NMSQuest). These are validated for use in PD and all include a section on gastrointestinal symptoms.

### 3.1. Upper Gastrointestinal Symptoms

Dysphagia in PD involves difficulty in the initiation and efficient completion of swallowing leading to decreased pace and comfort of eating and a reduction in quality of life [50,51]. Swallowing impairments might also contribute to malnutrition and weight loss and the occurrence of aspiration pneumonia constitute a major cause of death in PD [50].

Swallowing can be divided into an oral, pharyngeal, and esophageal phase. Generally, complaints of oropharyngeal dysphagia, e.g., difficulties swallowing or choking, are present in 35% of patients with a clear tendency to increase in prevalence and severity with disease progression [52]. Thus, marked dysphagia is often considered a late symptom of PD while severe dysphagia in early disease raises the suspicion of an atypical Parkinsonian disorder [2]. The presence of substantial dysphagia is not always reported by patients, but significant predictors of dysphagia include advanced clinical disease stage, drooling, significant weight loss, or body mass index below 20 [53]. Notably, drooling is a very common and troublesome feature of PD. However, it is not a consequence of hypersecretion of saliva, as the secretion is often decreased, but occurs when swallowing is impaired or infrequent causing accumulation of saliva in the mouth [54].

Oropharyngeal dysfunction includes inadequate mastication, poor formation of the bolus, difficulties in initiating swallowing, and choking as a sign of aspiration. As such, it has been considered as a motor symptom rather than a non-motor symptom. Accordingly, it often improves upon initiating medication [55] and it also improves during the on-state of medication even in the presence of dyskinesias [56,57]. Whether isolated esophageal dysfunction gives rise to distinct symptoms is unclear, although dysfunction of the lower esophageal sphincter might contribute to gastroesophageal reflux [52].

Symptoms attributed to gastroparesis are common in PD. Bloating and abdominal fullness has been reported by up to 50% of patients, while nausea and vomiting are reported by 15% [58,59]. Yet, rapid gastric emptying known as gastric dumping has also been reported [60]. Gastroparesis and gastric dumping are possible etiologies for unpredictable absorption of L-dopa with delayed onset of effect from anti-Parkinson medications as well as rapid effect resulting in dyskinesias.

Small intestinal bacterial overgrowth (SIBO) is a condition with increased bacterial density in the small intestines and has also been associated with disturbances in absorption and effect of anti-Parkinson medications. Furthermore, the condition is suspected to cause bloating, abdominal discomfort, and diarrhea [61]. However, these symptoms can also arise directly because of progressive neurodegeneration of the enteric and autonomic nervous system in PD, and the relative contribution from SIBO to the development of such symptoms is unclear.

### 3.2. Lower Gastrointestinal Symptoms

Infrequent bowel movements and straining during defecation are key symptoms of constipation [62]. Additionally, the perception of incomplete rectal emptying, abdominal discomfort, and pain that may be relieved by defecation are also attributable to constipation [63]. Studies of constipation are hampered by the lack of standardization and more than 10 different definitions of constipation have been applied in the PD literature alone [62].

The most frequently used definition of constipation is “less than 3 bowel movements per week,” which is used by the SCOPA-AUT and NMSS questionnaires, while “straining” alone is sufficient to fulfill the definition of constipation in the NMSQuest. A common feature of these widely used questionnaires is the aim of measuring the full burden of NMS in PD and not constipation in detail. Better suited for the latter is the Rome Functional Constipation questionnaires [63,64]. Although it has not been validated for use in PD, it provides a more detailed and quantifiable measure of constipation symptoms as is also the case with the Cleveland Constipation Scoring System [65].

A recent meta-analysis found that 40–50% of PD patients report less than 3 bowel movements per week compared to ~15% of matched controls [62]. However, the prevalence estimates in individual studies ranged from 8% to 70% in patients and from 0% to 34% in healthy controls underlining the questionable reliability associated with symptom-based investigations of constipation. This substantial variance is not only a consequence of different settings and questionnaires but possibly also aggravated by individuals slowly getting accustomed to symptoms as they develop over time. A significant recollection bias when reporting bowel movement frequencies as found by Ashraf et al. [66] may also contribute to the variance. Notably, a definition based purely on bowel movement frequency will also tend to overlook the presence of constipation if the patient suffers from co-existing diarrhea as seen when watery stools leak around a blockage of hard stool in cases of fecal impaction [67].

Interestingly, a meta-analysis has found that constipation in otherwise healthy adults increases the risk of subsequent PD diagnosis [5]. This association was present with an OR of 2.13 even in those patients whose constipation preceded the diagnosis of PD by more than 10 years [5]. The finding is supported by a more recent study of a large Danish cohort [21]. Similarly, constipation is frequent in RBD cases [68] and the prevalence is higher in PD patients with RBD than those without [69]. The causal mechanisms behind this association are unclear but might be related to pathological processes affecting the ENS and the DMV prior to recognizable loss of motor function.

Anorectal symptoms are very common in PD with straining being one of the most commonly reported gastrointestinal symptoms in PD. The prevalence of straining is ~70% in PD compared to around 40% in controls while incomplete emptying is reported by ~55% of patients and 28–42% of controls [19,20]. The considerable burden of anorectal symptoms in PD is further substantiated by the finding that 66% of early PD patients report defecatory symptoms, whereas only 29% reported a weekly bowel movement frequency of fewer than 3 times [70].

In summary, PD patients frequently suffer from a variety of gastrointestinal symptoms, although these symptoms remain difficult to define and measure using questionnaires. Consequently, objective measures are needed to assess functional disturbances of the gastrointestinal tract in order to advance our understanding of the underlying pathologies.

## 4. Objective Measures of Gastrointestinal Dysfunction

Gastrointestinal dysfunction can sometimes be subclinical, so measurable dysfunction is often more frequent than the corresponding subjective symptoms assessed by questionnaires. The following section covers the principles behind the most useful objective measures and summarizes key findings.

### 4.1. Swallowing Dysfunction

Successful swallowing involves complex voluntarily initiated movements followed by reflexes involving motor as well as sensory neurons of somatic and visceral origin [50,71]. As such, the basal ganglia are involved primarily in the oral and pharyngeal phase of swallowing during which the bolus is formed and by coordinated effort of striated muscles transported to the top of the esophagus [50,71]. The visceral fibers of the vagal nerve innervate the lower third of the esophagus [72]. The DMV and the vagal nerve are among the earliest and most severely affected structures in PD and show marked involvement in most patients [13]. LP has also been found in the peripheral pharyngeal nerve fibers [73,74] although the ambiguus nucleus innervating the pharyngeal muscles and the upper esophagus is relatively unaffected by LP [13,72]. Consequently, oropharyngeal and lower esophageal dysfunction are inherently different from a functional as well as a neuropathological perspective in the context of PD.

Oropharyngeal dysfunction can be visualized using fiberoptic endoscopic evaluation of swallowing (FEES) and by videofluoroscopic swallowing studies (VFSS). These methods are suitable for evaluating risk of aspiration in relation to different food consistencies and liquids being swallowed during visualization [50]. In PD, abnormalities during FEES are reported in as many as 95% of patients with residues being the most common finding (93%) but also with a significant finding of aspiration in 16% of asymptomatic PD patients [75].

These methods do not sufficiently evaluate esophageal dysfunction and thus, high-resolution manometry (HRM) compliments the use FEES and VFSS. HRM is performed by passing a thin pressure-sensitive tube through the nose to the stomach. Using HRM and FEES Suttrup et al. examined 65 PD patients of different disease stages and reported that 95% of cases had measurable impairments of esophageal motility [76]. These changes were seen almost evenly across all stages of disease and was without clear association to the FEES scores of oropharyngeal dysphagia [76]. Esophageal motility can also be evaluated by esophageal scintigraphy where a radioactively labeled bolus is swallowed during the dynamic recording of gamma emission. Using this principle Potulska et al. found significantly prolonged lower esophageal transit times suggestive of dysfunctional esophageal peristalsis in agreement with the findings of Suttrup et al. [77].

### 4.2. Stomach Dysfunction

Symptoms of gastroparesis are commonly associated with PD but the results from studies of objective gastroparesis are conflicting and methodological differences make individual studies difficult to compare [60].

**Figure 1:**
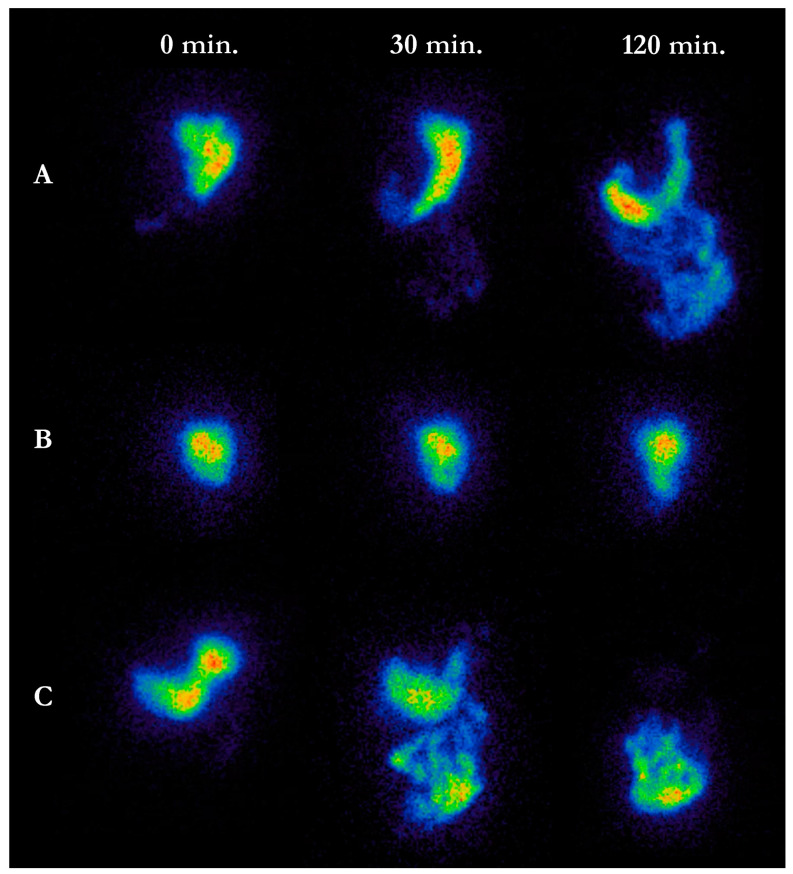
Gastric scintigraphy images at 0, 30 and 120 min after ingestion of a radioactive solid meal. (**A**). Healthy control with normal gastric emptying time. (**B**). Parkinson’s disease (PD) patient with severely delayed gastric emptying time compatible with gastroparesis. (**C**). PD patient with rapid gastric emptying suggestive of ”gastric dumping” (compare the image taken at 30 min. to the image from A taken at 120 min.).

Solid meal scintigraphy is considered the gold standard for estimating gastric emptying time (GET) [74]. After ingestion of a standardized meal containing ^99m^Tc the emitted gamma radiation is measured by serial images recorded by a gamma camera (Figure 1) [78].

A meta-analysis of studies using gastric scintigraphy in PD found a non-significant trend towards prolonged GET in PD patients [60]. However, the trend reached statistical significance after post-hoc exclusion of one outlier study.

Scintigraphy requires specialized facilities and exposes the patient to radiation but provides a reliable biomechanical measure of gastric emptying rate. Alternatively, a breath test using a meal containing ^13^C-sodium octanoate is an indirect measure of gastric emptying based on the subsequent measurements of expired ^13^CO_2_ [61]. This estimate is dependent not only on gastric emptying but also on small intestinal absorption and hepatic metabolism of the tracer [60]. This notion is supported by a comparative study of healthy individuals which found that the results of the breath test could not simply be adjusted to fit the results of the scintigraphy even though both methods are reproduciable within each subject [79]. Theoretically, small intestinal dysmotility and malabsorption, bacterial overgrowth, and changes in liver metabolism can all interfere with results of the breath test [60]. In this light, it is worth noting that most studies using the breath test reported prolonged GET in PD patients compared to controls [60].

Interestingly, the breath test has been used to study a broader spectrum of disease stages. Unger et al. found prolonged GET estimates in untreated PD but not in iRBD [80] whereas Epprecht et al. found no difference between early-stage PD patients in the off-state and controls [81]. Collectively, this suggests that the disturbances giving rise to pathological parameter estimates on breath tests are not a feature of prodromal PD but develops at later disease stages.

The GET may also be influenced by anti-Parkinson medications, and administration of levodopa to healthy individuals have been shown to delay GET [82,83]. A solid meal scintigraphy study by Hardoff et al. found no difference in GET between mild and moderate disease stage PD patients and likewise, no difference between treated and untreated PD patients [84]. However, studies investigating the association between GET and motor fluctuations in PD patients treated with levodopa have yielded conflicting results [84,85]. In a study using breath tests to compare GET before and 3 months following initiation of deep brain stimulation in the subthalamic nucleus (STN-DBS) found no significant difference when comparing the pre-operative, off medication condition with the pre-operative on medication condition—indicating that levodopa administration does not affect GET [86]. However, a marked decrease in GET was demonstrated in the post-postoperative on medication-on stimulation condition compared to the pre-operative on medication condition suggesting a positive effect of STN-DBS on gastric emptying.

Whether the heterogenicity in gastric emptying time is influenced by more distal dysfunction has not been investigated, but chronic rectal distension due to defecatory disturbances could induce a cologastric reflex causing a delay in GET. To our knowledge, this has only been demonstrated in individuals without PD [87,88].

Novel methods to study stomach dysfunction in PD have introduced new measures of dysfunction and shown promising results. An MRI-based imaging study observed decreased emptying of gastric volume in PD patients with early satiety and dyspepsia. Additionally, decreased total gastric volume and decreased gastric motility was reported in patients with dyspepsia [89]. Another study used an electromagnetic capsule system to study gastric motility in PD patients and found prolonged GET compared to controls but similar frequency of gastric contractions indicating normal functioning of the intestinal cells of Cajal [90]. These methods are yet to be validated in PD but offer the possibility of repeated measurements in the same individuals without exposure to radiation.

In summary, it is not possible to make firm conclusions about the frequency and magnitude of gastric dysmotility in PD, since the seemingly compelling results from ^13^C-sodium octanoate breath tests are prone to measuring other disturbances different from gastric emptying per se. However, the disturbances underlying these findings are noteworthy and further studies are needed to shed light on the association with symptoms and small intestinal dysfunction.

### 4.3. Small Intestinal Dysfunction

In comparison to the stomach and colon, very few studies have explored small intestinal dysfunction in PD.

Bacterial overgrowth of the small intestines is most often defined as above 10^5^ colony forming units (CFU) per milliliter of jejunal fluid acquired by endoscopic aspiration [61]. Alternatively, breath tests can demonstrate the presence of bacteria in small intestine by measuring the concentration of H_2_ in expired air following the intake of glucose or lactulose. Breath test provides a lower sensitivity (60–70%) and specificity (40–80%) when compared to jejunal aspiration [91] but are non-invasive and therefore used very frequently. However, the interpretation of breath test results is an area of ongoing discussion and the method is not fully validated [61].

In a study of PD patients, the prevalence of SIBO was investigated using both glucose and lactulose breath tests. Here, the SIBO prevalence was 54.5% in PD patients and 20% in controls with most cases being positive on only one of the two tests [92]. Another study used only glucose breath tests but reported a similar prevalence among PD patients and a prevalence of 8% among controls [93]. Interestingly, the former study found a higher frequency of delayed-on and increased daily off times [92] in patients with SIBO suggesting that SIBO could contribute to abnormal absorption of levodopa. Furthermore, a study by Tan et al. found the presence of SIBO to be associated with worse motor symptoms [94]. A possible contributing factor to this is the finding that small intestinal enterococcus species may inactivate levodopa via decarboxylases [95,96]. Along the same line, infection with *helicobacter pylori* (HP) is associated with worse motor symptoms [97,98] and in epidemiological studies, HP infection has been linked to development of PD later in life [99]. Substantially improved motor function has been observed after eradication [98,100], but a recent randomized, controlled trial of HP eradication in infected PD patients did not find any improvement of motor or nonmotor symptoms at weeks 12 and 52 following eradication [101].

Small intestinal transit has been investigated using different ambulatory systems comprised of an ingestible capsule and a wireless data receiver [102,103,104]. These studies reported a delay in small intestinal transit time in PD compared to matched controls, although the magnitude of delayed transit was less marked than that seen in the colon. In support, studies of colonic transit time which uses ingested radiopaque plastic markers (ROM) sometimes report the presence of ROM in the small intestine 24 h after ingestion of the last capsule [105]. Such findings are a clear indication that upper GI tract transit can be severely impaired in some PD patients.

### 4.4. Colonic and Anorectal Dysfunction

Mechanistically, constipation can be separated into slow transit constipation due to prolonged colonic transit time (CTT) and outlet obstruction caused by dyssynergia of rectal muscles [106]. Presumably, outlet constipation is related to anorectal symptoms such as straining and incomplete emptying while prolonged colonic transit may be closer related to decreased frequency of bowel movements.

**Figure 2:**
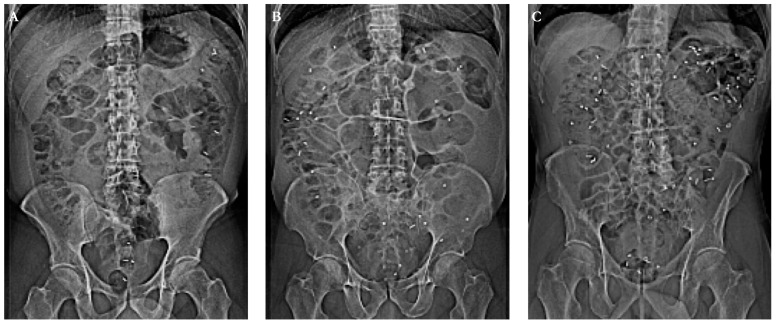
Abdominal x-ray topograms visualizing the retention of radio-opaque markers in the gastrointestinal tract as an objective measure of colonic transit time (CTT). (**A**). Healthy control with an estimated CTT within the normal range. (**B**). Parkinson’s disease (PD) patient with an estimated CTT near the mean for PD patients. (**C**). PD patient with severely prolonged CTT.

Objective measures of CTT are widely available and the most commonly used technique is based on the visualization of ingested radio-opaque markers (ROM) using abdominal x-ray (Figure 2) [107]. Typically, one capsule containing 10 ROMs is ingested for 6 consecutive days (a total of 60 markers) followed by an abdominal x-ray on day 7 revealing the number of retained markers. When this protocol was used with a cut-off of 25 markers for males and 29 markers for females, 80% of Parkinson’s patients had prolonged CTT [105]. Specifically, the retention of markers is predominantly in the rectosigmoid part of the colon suggesting that outlet obstruction is a substantial contributor to the finding of CTT in PD [108,109]. Importantly, the correlation between objectively prolonged CTT and subjective symptoms as measured by questionnaires is generally poor [105,110,111]. Notably, the frequency of bowel movements seems to be a worse predictor of prolonged CTT than other symptoms such as bloating and use of an enema or manual evacuation of feces [109].

Similarly, a study using a magnetic 3D-Transit system found no correlation between constipation and neither small intestinal nor colonic transit times, although both measures of intestinal transit were prolonged in the PD group compared to controls [102]. Evaluation of total and regional colonic volumes is possible when an abdominal CT-scan is performed, and with this method increased total colonic volume was demonstrated in a group of 22 iRBD cases compared to 26 controls [68]. Notably, the difference in total colonic volume was statistically stronger than the corresponding difference in colonic transit times as measured by radio-opaque markers as well as magnetic 3D-Transit capsule. The robustness of colonic volumetric measures was also utilized in a study comparing newly diagnosed PD patients. Here, a highly significant increase in colonic volume was detected in PD patients with RBD when comparing to PD patients without RBD [45].

Anorectal dysfunction in isolation or in combination with prolonged CTT is probably a major contributor to constipation in PD. Several approaches have been utilized to study different aspects of anorectal dysfunction including defecography, electromyography (EMG), balloon distension tests, and rectal manometry. Generally, studies of anorectal dysfunction in PD have used heterogeneous methods, small sample sizes, and often without control groups. In brief, incomplete emptying with dysfunction of the puborectalis muscles and paradoxical contraction of the external anal sphincter or lack of inhibition has been demonstrated by defecography and manometry, respectively [112,113]. Paradoxical sphincter contraction on defecation together with incomplete emptying have also been demonstrated in another study using rectoanal videomanometry [108]. Rectal sensitivity of urge was found to be normal in a study of unselected PD patients [113,114], while another study points to the possibility of rectal hypersensitivity in constipated PD patients [110]. Balloon expulsion tests in 35 PD patients, who did not fulfill the ROME-III criteria for defecatory dysfunction (DD), demonstrated abnormal expulsion in 27 of 35 cases compared to 24 of 35 in otherwise healthy adults fulfilling the criteria for DD [115]. Once again, these findings highlight the often poor correlation between subjective symptoms and objective measures [62]. Interestingly, no differences were found on manometry between early and late PD patients suggesting that significant dysfunction is present early in the disease [115]. In support, another study used manometry and reported a similar prevalence of pelvic floor dyssynergia of approximately 60% in early as well as in late PD [114].

**Figure 3:**
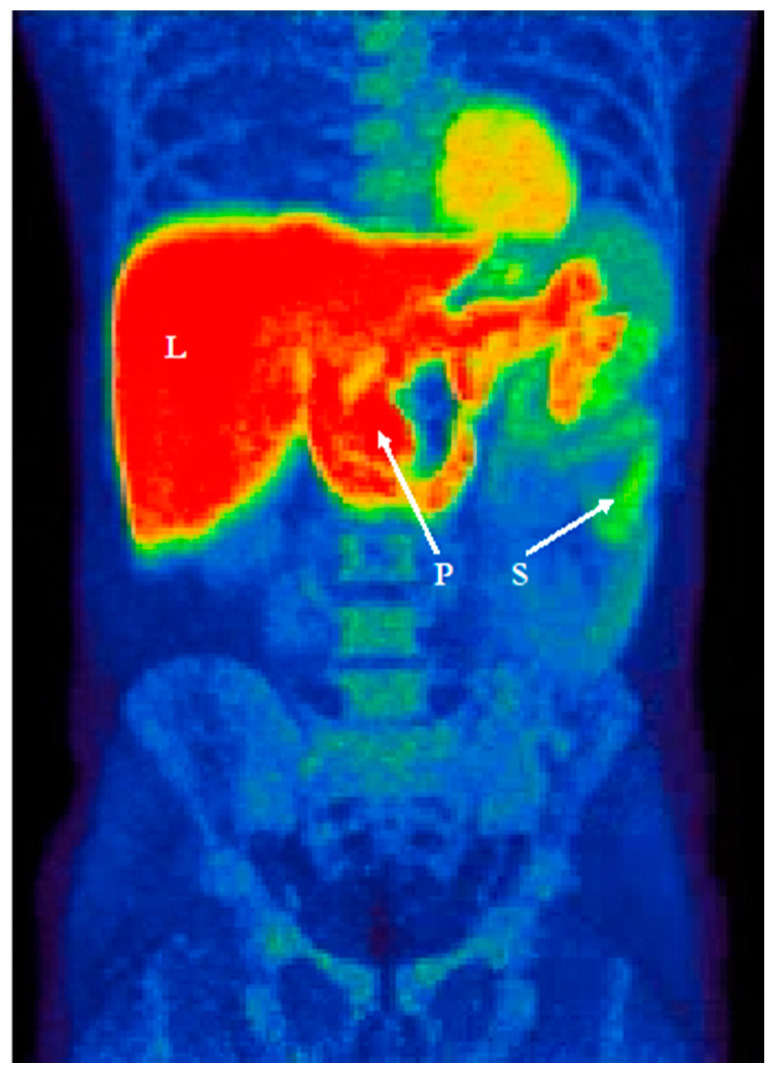
^11^C-Donepezil PET illustrating the summed signal of gastrointestinal organs (L liver, P pancreas, S small intestine).

Recent studies used ^11^C-donepezil PET/CT (Figure 3) to measure cholinergic denervation in the GI tract of PD patients and reported decreased cholinergic signal in the small intestine and particularly in the colon [116,117]. Interestingly, similar magnitudes of decreased colonic signal are seen in iRBD patients, suggesting that cholinergic denervation is already manifest in the prodromal phase [44]. Although 70% of enteric neurons are cholinergic, the loss of cholinergic PET signal in the intestines is best compatible with parasympathetic denervation, since it is known that the DMV shows severe pathology and cell loss in PD, whereas no significant loss of enteric neurons has been detected [118].

It is well documented that PD patients show very dramatic sympathetic denervation of the heart [119,120]. Nearly all iRBD patients show the same profound loss of cardiac sympathetic signal, signifying that this subtype of prodromal PD show involvement of the autonomic system before the brain is markedly affected [45,121,122,123]. However, the importance of sympathetic denervation for gastrointestinal dysfunction is presently unclear and no studies have documented sympathetic denervation of the intestines in PD.

## 5. Treatment of Gastrointestinal Symptoms in PD

Recent review papers have provided detailed recommendations for treatment of gastrointestinal symptoms in PD [124,125]. Thus, treatment strategies will only be briefly summarized here.

For the treatment of drooling, behavioral modifications such as chewing gum have been suggested [54] as this may increase the rate of swallowing. An anticholinergic such as glycopyrrolate may give or exacerbate constipation and urinary retention, and local treatment options including oral atropine solutions and hyoscine patches as well as parotid and submandibular botulinum toxin injections are therefore often favored [124,125]. Other medications with anticholinergic properties might also be useful although evidence of their efficacy is limited.

For oropharyngeal dysphagia, the positive effects of optimized anti-Parkinson treatment on symptom severity is well established [55,56,57]. If symptoms persist, a speech and language therapist may initiate swallowing treatment with the use of methods aimed at the individual patient’s difficulties—often based on objective evaluations such as fiberoptic endoscopic evaluation of swallowing [50].

Treatment of gastroparesis in PD is complex as the diagnosis cannot be made from symptoms alone and since pharmacological treatment is associated with a substantial risk of adverse effects. The prokinetic dopamine receptor antagonist domperidone is possibly useful for treatment of nausea and delayed gastric emptying in PD, since it does not cross the blood-brain barrier in contrast to metoclopramide [124]. Future treatment options might include the use of a gastric pacemaker as this has shown promising results in scintigraphy-confirmed gastroparesis caused by diabetic neuropathy [126].

SIBO is treatable with antibiotics and can lead to a reduction in motor fluctuations in some patients [61,92]. Eradication of SIBO using empirical antibiotic treatment has been demonstrated in populations without PD although recurrence rates of up to 44% after 9 months have been reported [61]. Theoretically, antibiotic treatments of SIBO impose a risk of generating resistant gastrointestinal infections and disturbance of colonic microbiota [61]. Clearly, there is a need for further studies evaluating the effects of SIBO eradication in PD.

For constipation, lifestyle modifications such as exercise and gradually increased fiber and fluid intake are often advised for the general population with functional constipation [127]. Although this has not been specifically investigated in PD patients, it is often recommended for this population as well [124]. Several studies have investigated the effects of pharmacological treatments of constipation in PD [124] and support the use of the bulk-forming psyllium [110], PEG (Macrogol) containing osmotic laxatives, and the chloride channel activator Lubiprostone [124]. Recently, a randomized controlled trial investigated the effects of a daily capsule containing a multi-strain probiotic supplement in PD patients and demonstrated an increase in spontaneous bowel movements in the treated group [128]. Furthermore, the patient-reported treatment satisfaction was 65.6% in the treated group compared to 21.6% in the placebo group supporting not only the feasibility of probiotic supplements in PD but also the possible interconnection between the microbiome and constipation. Specifically aimed at outlet constipation, biofeedback therapy has shown promising results in other patient populations [129,130] and ultimately, botulinum toxin injections into the puborectalis muscle have been found to be effective in PD patients with outlet constipation [131,132].

## 6. Conclusions

In conclusion, subjective gastrointestinal symptoms are common in PD and the prevalence of objectively measured dysfunction is even higher. Oropharyngeal dysphagia is often asymptomatic during the early stages, and since it improves with levodopa treatment, it is often viewed as a motor symptom. The prevalence and magnitude of delayed gastric emptying is unclear since findings in solid meal scintigraphy studies indicate that gastric emptying is only marginally delayed in early-to-moderate stage PD. Breath test studies generally report a more significant delay in gastric emptying of PD patients, but further studies are needed to clarify the extent to which small intestinal dysfunction and perturbed liver metabolism contribute to these observations.

Small intestinal bacterial overgrowth and altered microbiome in PD patients are active fields of investigation and highlight the complex interplay between microbiota and gastrointestinal dysfunction. Constipation is among the most common non-motor symptoms in PD, but research in this field is hampered by a lack of standardization and the symptoms of anorectal dysfunction are often missed. Additionally, the prevalence of objective colonic dysfunction in terms of delayed colonic transit and anorectal dysfunction far exceeds the reported frequency of subjective constipation and indicates that the gut is affected in the vast majority of PD patients.

Looking forward, studies of prodromal cases such as those with iRBD may provide important insights into the sequence of events behind the development and progression of PD. Additionally, further clinical trials are needed that specifically test tailored treatments of gastrointestinal symptoms in well characterized groups of PD patients.

## Data Availability

Not applicable.
